# A Peculiar Case

**Published:** 1869-12

**Authors:** S. L. Bracy


### A PECULIAR CASE.

BY S. L. BRACY.

A lady about 35 years of age, dark hair and eyes, large and very fleshy, rather below the medium hight; called to have a tooth extracted; upon examination, I found the root of the eye tooth on the right side the only one remaining in the mouth (all of the others having been extracted six weeks before), and that broken off even with the gums and considerably inflamed about it. After cutting around it well, so as to get a fixed hold, it was extracted with very little difficulty, though quite painful. As soon as extracted, I saw the blood flow freely from the right nasal cavity. She observing, that something had been broken or torn loose between her nose and tooth, or mouth. As she felt the cold air pass from the nose through the alveolar cavity (from whence the tooth came) into the mouth, in an instant almost; however, after the root had been extracted the parts surrounding the orifice closed in, so that no more air passed through that orifice. The blood continued to flow freely from the right nasal cavity for about five minutes, and then ceased. There was very little blood from the alveolar cavity into the mouth. I then examined the root which I found about medium size and length. Just at the point appeared to be partially absorbed, or sharpened, and the point enclosed in a sack or abscess. After the bleeding ceased, I examined the mouth, found nothing unusual in appearance, but on introducing a probe, I found the opening passed through from the mouth into the nasal cavity, and the probe passed into the nose and produced a disposition to cough and spit, as if something had lodged in the cavity of the nose. There was no other difficulty or inconvenience, the wounds healing up very well and rapidly, and in a few days was without soreness or inflammation.

The lady informed me that when the corresponding tooth of the left side was extracted, she experienced a similar feeling, that is, felt as if something had been broken or torn loose between the point of the root and nose, but no blood came from the nose, nor was there any air passed from the nose to the mouth through the alveolar cavity, but says that the cavity of the nose on that side feels considerably larger to her than it did previous to the extraction of the tooth. She has experienced no trouble or inconvenience since the extraction, except some dull pain in the face and jaws for a day or so subsequent to the extraction.

I have never met a similar case, nor did I ever hear of one, would be glad of an explanation, whether or not it is common.

